# Effect of Feeding a Calcium Chloride Supplement on Sow Stillbirth Rate

**DOI:** 10.3390/ani14030516

**Published:** 2024-02-04

**Authors:** Sahara Craig, Si-En Ruth Khaw, Kiro R. Petrovski, Roy N. Kirkwood

**Affiliations:** 1 School of Animal and Veterinary Sciences, University of Adelaide, Roseworthy Campus, Roseworthy, SA 5371, Australia; sahara.craig@student.adelaide.edu.au (S.C.); si-enruth.khaw@adelaide.edu.au (S.-E.R.K.); 2 Davies Livestock Research Centre, School of Animal and Veterinary Sciences, University of Adelaide, Roseworthy Campus, Roseworthy, SA 5371, Australia; kiro.petrovski@adelaide.edu.au

**Keywords:** dietary manipulation, serum calcium, urine pH, farrowing

## Abstract

**Simple Summary:**

In the present study, we tested whether supplementing dietary calcium for older sows would increase their blood calcium concentrations and decrease the incidence of stillbirths. We found that the increased dietary calcium did not influence blood calcium concentrations or the incidence of stillbirths. However, regardless of treatment, sows with low levels of calcium were associated with an increased incidence of stillbirths. In order to minimize stillbirths, sow nutritional management must aim to maintain adequate serum calcium concentrations.

**Abstract:**

The present study was undertaken to determine the effect of daily calcium chloride (CaCl_2_) supplementation from day of entry into the farrowing house until day of farrowing (6.4 ± 0.3 d) on stillbirth rates. Landrace × Large White sows (parities 4 to 6; *n* = 53) were offered 40 g/d CaCl_2_ (*n* = 28) or served as controls (*n* = 25). The morning before their estimated farrowing date, a blood sample was obtained from 25 sows for calcium measurement and a urine sample from 22 sows for pH measurement. The feeding of CaCl_2_ decreased urinary pH compared to the control group (*p* < 0.001), indicative of an induced metabolic acidosis, but there was no effect of feeding CaCl_2_ on serum calcium concentrations or the incidence of stillbirths. Nonetheless, regardless of treatment, sows with higher serum calcium concentrations (>2.5 vs. <2.5 mmol) or lower urine pH (<7.0 vs. >7.0) had fewer stillborn piglets (*p* < 0.001 for both). While showing that low serum calcium levels will increase stillbirth rates, our data indicate that the administration of 40 g/d CaCl_2_ for 6 d prior to farrowing was not sufficient to increase serum calcium or decrease stillbirth incidence.

## 1. Introduction

Foetal losses pose a significant concern for the pig industry, presenting both welfare and economic challenges within commercial swine herds [1]. Stillbirths are primarily attributed to non-infectious causes such as prolonged farrowing durations and associated intrauterine asphyxia [2]. Stillbirths can affect 40% or more of all litters and account for almost 8% of all piglets born [1,3]. Notably, these percentages increase when considering higher-parity sows [4], presumably due to their larger litters and longer farrowings. Calcium ions play a crucial role in regulating muscle contractility, including the myometrium [5], with contractions induced and prolonged by an increased calcium concentration [6]. With larger litter sizes and increased milk yield being the requirements of modern sow genotypes, sows have a greater demand for calcium [7]. Interestingly, supplementing calcium ions in feed significantly reduces the number of stillbirths and reduces the average farrowing duration of young sows [8]. Although hypocalcaemia would be expected to adversely impact myometrial contractility, there is a dearth of data related to sow serum calcium concentration in the period encompassing parturition.

Calcium chloride (CaCl_2_) is known to be an acidogenic agent leading to metabolic acidosis, a phenomenon well documented in dairy cows. The CaCl_2_ induces a shift in the electrolyte composition of the diet resulting in a decrease in the dietary cation–anion difference (DCAD), calculated as the difference between positively charged strong cations (sodium and potassium) and negatively charged strong anions (chloride and sulphur) in the diet: $DCAD=\left(\left[Na^{+}\right]+\left[K^{+}\right]\right)-(\left[Cl^{-}\right]+\left[S^{2-}\right])$. Negative or positive DCAD values indicate a more acidogenic or alkalogenic diet, respectively [9]. Dietary supplementation of sows with acidogenic minerals is expected to yield a negative DCAD, thus contributing to metabolic acidosis [10,11] and subsequently resulting in parathyroid activation. The release of the parathyroid hormone (PTH) enhances calcium resorption from the bone and increases absorption in the small intestine, resulting in hypercalcaemia and excess ionized calcium in the body. Interestingly, while calcium mobilization was evident, Guo et al. (2019) [11] found no difference in vitamin D or parathyroid concentrations during lactation. The surplus calcium is excreted in the urine, leading to aciduria and calciuria [12]. The higher milk yields in multiparous sows likely result in a greater need for calcium resorption from bones. We hypothesized that providing supplemental dietary CaCl_2_ to older sows would induce physiological hypercalcaemia, or otherwise correct hypocalcaemia, and result in an aciduria indicative of a metabolic acidosis. This would be evidenced by decreased stillbirths.

## 2. Methods

### 2.1. Selection and Treatment

The present study was conducted at the University of Adelaide Roseworthy piggery with the approval of the University of Adelaide Animal Ethics Committee (application 37187, approved 25 March 2023). During the study, sows were housed in conventional farrowing crates. From the day of their entry into the farrowing house until the day of farrowing 6.4 ± 0.3 d later, 53 Landrace × Large White sows (parities 4 to 6) were fed 2.5 kg/d, over two feeds (at 0700 and 1400 h), of a lactation diet commercially formulated to provide 14 MJ DE/kg, 17.8% protein, 1.05% lysine, and 0.3% dicalcium phosphate. Water was available ad libitum from nipple drinkers. Sows were assigned by parity to either be offered 40 g/d supplemental dietary CaCl_2_ mixed into their first daily feed (NutriCAB, Kemin Australia, Killara, NSW, Australia; *n* = 28) or to serve as controls (*n* = 25). The CaCl_2_ dose was based on a pilot study where sows were offered 140 g/d but feed refusals were observed, which was also seen when feeding 80 g/d. Feed refusals were not evident when 40 g/d CaCl_2_ was offered. Farrowing induction was not employed. The morning before their estimated 115 day farrowing date, a 3 mL blood sample was obtained via a prominent auricular vein into a serum separation tube from 13 CaCl_2_-fed and 12 control sows, centrifuged, and the serum stored at −20 °C until assayed for total calcium concentration using a calcium Arsenazo photometric colour test (Beckman Coulter Australia, Mount Waverley, VIC, Australia). No additional blood samples were taken to minimize sow stress. Additionally, a mid-stream free-catch urine sample was obtained from 11 sows per treatment for pH determinations using a commercial urine dipstick (Combur^10^ Test^®^ UX, Roche, Bayswater, VIC, Australia). Sows sampled were those who urinated serendipitously during a 2 h observation period after the first daily feed. The total numbers born and born alive from all farrowings were recorded.

### 2.2. Statistical Analyses

All statistical analyses were performed using SAS version 9.4 (Statistical Analysis Software, Cary, NC, USA). There were 365 sow-related data point observations available for analysis. After bootstrapping the data at a root of 10 possibilities per sow ID, 2261 sow observations were available for analysis. Bootstrapping was carried out to decrease the standard errors. All data were analysed before and after bootstrapping.

- Blood calcium was assigned to one of two categories: low (<2.50 mmol/L) or high (≥2.50 mmol/L)
- Urine pH was assigned to one of two categories: low (<7.0) or high (≥7.0)

The cut-off values were arbitrary. The effects of the calcium feeding (CaCl_2_ or control) on the variables of piglets born alive, parity, serum calcium, stillbirths, and total litter size were estimated using a mixed model in PROC MIXED, as presented in the following equation:

$$Dependent\;variable={Treatment}_{Month\;of\;farrowing}+{Residual}_{Month\;of\;farrowing}$$

where month of farrowing is replicated (*n* = 2). The preliminary model tested the effect of litter size, parity, room of farrowing, and sow on the outcome of interest (dependent variable), but these were not significant and were thus excluded from the final model. The outcomes of the model were the least square means and their respective standard errors.

## 3. Results

The similarity in mean values between the raw and bootstrapped data confirmed that the final results were not biased. As shown in Table 1, no differences between treatments were observed for most of the dependent variables, except in the number of piglets per litter (*p* = 0.02) and the urinary pH (*p* < 0.001). The effect of CaCl_2_ supplementation to sows pre farrowing was evident in the urine pH, but despite evident aciduria, there was no effect on serum calcium concentrations or stillbirth rates.

**Figure 1:**
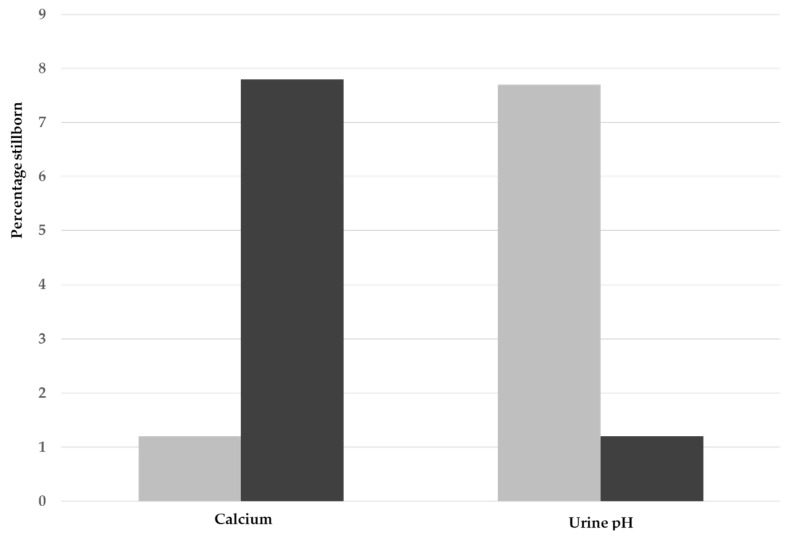
Effect of high (grey bars) and low (black bars) concentrations of serum calcium (low < 2.50 mmol/L; high ≥ 2.50 mmol/L) and urine pH (low < 7.0; high ≥ 7.0) on the proportion of stillborn piglets per litter for the bootstrapped data.

Independent of treatment, the effects of serum calcium concentrations and urine acidification on the proportions of stillbirths are summarized in Figure 1. The effects of the calcium and urine categories on the proportion of stillborn piglets was highly significant, with high stillbirth rates associated with low serum calcium and higher urine pH (*p* < 0.001 for both).

## 4. Discussion

In the present study, we observed no effect of feeding 40 g/d CaCl_2_ on the number of stillborn piglets. This contrasts with an unpublished preliminary study where 140 g/d was fed to sows and stillbirth rates were reduced. However, in that study, there were significant feed refusals, so the actual consumption remains unknown. Prior to the present study, we determined that 40 g/d would be consistently eaten, with no feed refusals noted. We did detect a significant difference in urinary pH between the CaCl_2_-fed and control sows, with the CaCl_2_-fed sows having a lower pH, indicating metabolic acidosis [8,13]. This was expected, as CaCl_2_ is an acidogenic agent and causes metabolic acidosis in pigs [8,14]. The feeding of CaCl_2_ causes a negative DCAD diet [13], the results of which have been studied in dairy cows at great length [9,15] and, presumably, may be applicable to sows [16]. A lower DCAD results in a net gain of acid [8] and the state of metabolic acidosis triggers the release of the parathyroid hormone [17]. This, in turn, increases the resorption of calcium from bones and upregulates the absorption of calcium in the small intestine [18]. When calcium levels are in positive balance due to supplementary dietary calcium, the excess calcium is excreted in urine, which is associated with aciduria.

Interestingly, even though supplementing sows with CaCl_2_ resulted in a low urine pH of 5.3, indicating the successful induction of a metabolic acidosis, the supplemental calcium did not affect serum total calcium concentrations. This is in contrast to previous data indicating an increase in both total and ionised calcium in sows following feeding with increased dietary calcium from 86 days of gestation [19]. Ionised calcium normally exists in equilibrium with complexed and protein-bound calcium in extracellular fluid and a decrease in serum pH should increase the ionised calcium concentration [18]. In the CaCl_2_-supplemented sows, despite having similar serum total calcium levels, the low urine pH suggests a probable metabolic acidosis and so it is likely that the ionised calcium was higher. However, in the absence of appropriate measurements, this suggestion remains speculative.

Calcium supplementation did not have a significant effect on the number of piglets stillborn, which may be a result of the 6-day feeding period being too short. Indeed, while negative-DCAD diets are very effective in dairy cows, clinical experience suggests a feeding period of at least 10 days is required, and previous sow studies began feeding by about 2 to 3 weeks pre farrowing [10,11]. In addition, the dose of 40 g/d of calcium we supplied may have been too low, with others finding efficacy when feeding 70 g/d [8]. However, our sows tended to refuse to consume higher doses. Alternatively, any effect of supplemental calcium could be influenced by the current individual calcium status, with the correction of occasional underlying hypocalcaemia having a positive effect. Further, the timing of the feedings could also have affected the efficacy of the supplement, as afternoon feeding (1500 h) has been shown to result in a significant reduction in stillbirths compared to morning feeding [7].

There are many other factors that increase the risk of stillbirths, including higher parities, larger litter sizes, and the level of farrowing supervision [20]. In our experiment, parities were not different between groups. Further, while the difference in litter sizes was statistically significant, the biological significance of this small difference is arguably negligible. The level of supervision was similar across groups; although, due to the lack of supervision outside of staffed hours, sows that farrowed during unsupervised times were potentially at a higher risk of experiencing stillbirths. This was not recorded and could have influenced the results.

In contrast to cattle, where providing excess calcium pre calving may induce hypocalcaemia [21], the provision of excess calcium to sows in late gestation has not been shown to result in hypocalcaemia [19]. Therefore, if considering the supplementation of calcium to sows, there would appear to be no harm in excessive supplementation other than cost. Future research efforts should be made towards finding out the minimum level required to be efficacious.

Although the calcium supplementation did not give rise to a significant difference in the numbers of stillbirths in this study, the physiological effect indicated by the aciduria is promising for future research. It is of interest that an effect was evident in the prior unpublished pilot study with a potentially higher, albeit unknown, CaCl_2_ intake, with stillbirths being reduced by 50%. Previous studies have reported that lowering the DCAD of diets also has positive effects on farrowing kinetics, with effects on sows that last up to weaning [22] and into their subsequent farrowing [23]. To maximise intestinal absorption of a dietary calcium supplement, lowering the DCAD for 10 days before parturition could be a potential method of successfully reducing stillbirths. Of particular interest, and in support of our underlying hypothesis, when treatment was ignored, a decrease in stillbirths was recorded for the sows that had high serum calcium compared to those with low serum calcium, with a similar effect noted in the prior pilot study, highlighting the importance of serum calcium levels at parturition. Future research should consider if this was an effect of the calcium supplementation or the DCAD by using various acidogenic dietary additives.

## 5. Conclusions

This study has shown that the supplementation of 40 g/d CaCl_2_ for 6 days was insufficient to reduce stillbirths. A limitation of our study is that we provided the CaCl_2_ in order to manipulate the dietary DCAD, but we did not determine the actual DCAD value of the diets fed. However, the supplement did have a physiological effect, as indicated by the aciduria in the treated sows. Future research should be conducted looking at the time of feeding, the duration of feeding, and the supplement dose. While this study has shown that there is a potential detrimental effect of low serum calcium levels leading to increased stillbirth rates, further research is needed to fully assess this relationship.

## Figures and Tables

**Table 1:** Least square means ($\bar{x}$) and their respective standard errors (SEs) per average sow of variable parameters for the effect of calcium chloride (CaCl_2_) feeding at 40 g per day in the morning pre farrowing, adjusted for the month of farrowing and total litter size for the bootstrapped data. Serum calcium = serum calcium level of the sow, measured on the morning of the estimated due date; Stillbirths = total number of stillborn piglets per sow; Urinary pH = urine pH measured in a mid-stream free-catch urine sample taken within 24 h before farrowing.

Parameter	Control		CaCl_2_		*p*-Value
	N	$\bar{x}$ ± SE	N	$\bar{x}$ ± SE	
Parity	151	4.4 ± 0.2	178	4.1 ± 0.2	0.26
Piglets born alive	151	12.3 ± 0.2	178	12.4 ± 0.2	0.92
Stillbirths	151	1.2 ± 0.2	178	1.2 ± 0.2	0.86
Serum calcium (mmol/L)	75	2.5 ± 0.2	80	2.6 ± 0.2	0.70
Urinary pH	70	6.5 ± 0.2	62	5.2 ± 0.2	<0.001

## Data Availability

Data are contained within the article.
